# Molecular characterization of extended spectrum β -lactamases enterobacteriaceae causing lower urinary tract infection among pediatric population

**DOI:** 10.1186/s13756-018-0381-6

**Published:** 2018-07-28

**Authors:** Nahla O. Eltai, Asmaa A. Al Thani, Khalid Al-Ansari, Anand S. Deshmukh, Eman Wehedy, Sara H. Al-Hadidi, Hadi M. Yassine

**Affiliations:** 1 Biomedical Research Center, Qatar University, P.O. Box 2713, Doha, Qatar; 2 Pediatrics Department, Hamad Medical Corporation, Doha, Qatar; 3 Department of Laboratory Medicine and Pathology, Hamad Medical Corporation, Doha, Qatar; 4 College of Health Sciences, Qatar University, Doha, Qatar

**Keywords:** Urinary tract infection (UTI), Enterobacteriaceae, Antibiotic resistance, Children, ESBL, Qatar

## Abstract

**Background:**

The β-lactam antibiotics have traditionally been the main treatment of Enterobacteriaceae infections, nonetheless, the emergence of species producing β- Lactamases has rendered this class of antibiotics largely ineffective. There are no published data on etiology of urinary tract infections (UTI) and antimicrobial resistance profile of uropathogens among children in Qatar. The aim of this study is to determine the phenotypic and genotypic profiles of antimicrobial resistant Enterobacteriaceae among children with UTI in Qatar.

**Methods:**

Bacteria were isolated from 727 urine positive cultures, collected from children with UTI between February and June 2017 at the Pediatric Emergency Center, Doha, Qatar. Isolated bacteria were tested for antibiotic susceptibility against sixteen clinically relevant antibiotics using phoenix and Double Disc Synergy Test (DDST) for confirmation of extended-spectrum beta-lactamase (ESBL) production. Existence of genes encoding ESBL production were identified using polymerase chain reaction (PCR). Statistical analysis was done using non-parametric Kappa statistics, Pearson chi-square test and Jacquard’s coefficient.

**Results:**

201 (31.7%) of samples were confirmed as Extended Spectrum β -Lactamases (ESBL) Producing Enterobacteriaceae. The most dominant pathogen was *E. coli* 166 (83%) followed by *K. pneumoniae* 22 (11%). Resistance was mostly encoded by ^*bla*^ CTX-M (59%) genes, primarily ^*bla*^ CTX-MG1 (89.2%) followed by ^*bla*^ CTX-MG9 (7.7%). 37% of isolated bacteria were harboring multiple ^*bla*^ genes (2 genes or more). *E. coli* isolates were categorized into 11 clusters, while *K. pneoumoniae* were grouped into five clonal clusters according to the presence and absence of seven genes namely ^*bla*^ TEM, ^*bla*^ SHV, ^*bla*^ CTX-MG1, ^*bla*^ CTX-MG2, ^*bla*^ CTX-MG8 ^bla^ CTX-MG9*,* ^*bla*^ CTX-MG25.

**Conclusions:**

Our data indicates an escalated problem of ESBL in pediatrics with UTI, which mandates implementation of regulatory programs to reduce the spread of ESBL producing Enterobacteriaceae in the community. The use of cephalosporins, aminoglycosides (gentamicin) and trimethoprim/sulfamethoxazole is compromised in Qatar among pediatric population with UTI, leaving carbapenems and amikacin as the therapeutic option for severe infections caused by ESBL producers.

## Background

*Enterobacteriaceae* carrying extended-spectrum β-lactamases (ESBLs) is a global concern that demands global attention due to the limited available treatment options [1–4]. Over the past two decades, there has been an exponential increase in β-lactamase resistance worldwide accompanied with a significant escalation in the prevalence of ESBL-producing Enterobacteriaceae [5]. Unfortunately, ESBL-producing bacteria in children have come to the forefront of emerging antibiotic-resistant bacteria worldwide [6]. β-lactamases are divided into four functional groups: penicillinases, ESBLs, carbapenemases, and AmpC-type cephalosporinases [7]. Specifically, ESBLs is a group of plasmid-encoded enzymes that confer resistance to third generation cephalosporins [8–10]. ESBLs are divided into three groups according the encoding of TEM, SHV and CTX-M genes [11, 12]. CTX-M enzymes are the most common and are further classified into five major phylogenetic groups based on gene sequences namely, CTX-M -1, CTX-M-2, CTX-M-8, CTX-M-9, and CTX-M-25 [11]. ESBL-producing *Escherichia coli (E. coli*) and *Klebsiella pneumoniae* (*K. pneumoniae*) are the predominant organisms in childhood infections, and they pose significant threat to human health [13]. These organisms are listed among the pathogens for which there are few potentially effective drugs [1]. About 57% of bloodstream infections are caused by ESBL-producing Enterobacteriaceae, which are more likely to result in death compared to the infections caused by a non ESBL-producing strains [14].

Urinary Tract Infections (UTIs) continue to be one of the most common cause of illness in young children worldwide [9]. It distresses the child, concern the parent, and may cause permanent renal sequelae. The β-lactam antibiotics have traditionally been the main antibiotics for treatment of infections caused by Enterobacteriaceae, but the emergence of ESBL has rendered this class of antibiotics largely ineffective. It is therefore important to run epidemiological studies to define the epidemiology and profiles of these resistant bacteria for its impact on the development and implementation of stewardship programs.

High prevalence of extended-spectrum-β-lactamase (ESBL) and carbapenemase producing gram negative bacteria (GNB) has been reported in the Arabian Peninsula [15]. Nonetheless, little is known about the prevalence and profile of these bacteria in pediatric population [16] in the region. Prevalence of ESBL in urine among pediatric population has been gradually increasing in Qatar (HMC annual antibiogram). For example, the percentage of *E.coli* ESBL producers have gradually increased from 18% in 2010 to 24% in 2014, and reached 31.7% in 2017 (January–June) as reported in this study.

This is the first study that describes at the molecular level the genotypic profile of ESBL producing bacteria among children with UTIs in Qatar. Our data indicates rapid increase in ESBL resistance among Enterobacteriaceae in pediatrics with UTI, which mandates rapid regulatory and monitoring reforms at the State level.

## Methods

### Clinical isolates and controls strains

Ethical approval for this study was obtained from the Medical Research Centre (MRC), Hamad Medical Corporation (HMC), Doha, Qatar, protocol no. 16434/16. A total of 727 urine Samples were collected between February and June of 2107 from children (0–15 years of age) hospitalized with lower UTI at the Pediatric Emergency Center-HMC. All urine analysis were performed on patients presented with symptoms, mainly fever and dysuria. Urinary catheter was applied for all patients less than or equal 2 years of age, cerebral palsy (CP) patients and patients under intermittent catheterization. Otherwise, urine was obtained from mid-stream catch. Samples that did not yield significant bacterial growth, those that had multiple organisms and samples with suspected contamination as per lab report, were excluded from the study, and no duplicate samples were collected. All of the reported cases had UTI as their primary diagnosis. For each patient, demographic data such as age, nationality, and gender were collected. Out of the above samples, 635 (87.3%) were positive for Enterobacteriaceae species which were then isolated using readymade Cystine Lactose Electrolyte-Deficient media (IMES, Doha, Qatar). Isolated bacteria were identified by MALDI-TOF (Bruker Daltonik GmbH, Leipzig, Germany) and initial antimicrobial susceptibility testing was performed by Phoenix using the NMIC/ID-5 panel (BD Biosciences, Heidelberg, Germany) according to the manufacturer’s recommendations. Both automated tests were performed at Hamad General Hospital Microbiology laboratory. All intermediate resistant isolates were considered as susceptible. Initial testing with Phoenix revealed 201 (31.7%) isolates as Extended Spectrum β -Lactamases (ESBL) producer Enterobacteriaceae. Susceptibility testing was done for 16 clinically relevant antibiotics. 110 of these samples were randomly selected for further genotypic analysis.

Standard strains, *E. coli* ATCC® 25,922 and *E. coli* ATCC® 35,218, were used as controls for antimicrobial drug susceptibility testing. *E. coli* NCTC® 13,461™, *E. coli* NCTC® 13,462™, *E. coli* NCTC ®13,463™, *Enterobacter cloacae* NCTC ®13,464™ and *K. pneumonia* NCTC® 13,465™, *E. coli* ATCC® 35,218™ and *E. coli* NCTC ®13,368™ were used as positive controls, for CTX-M G1, CTX-M G 2, CTX-M G 8, CTX-M G 9, CTX-M G25 25, ^*bla*^ TEM and ^*bla*^ SHV, polymerase chain reaction (PCR) assays, respectively.

### ESBL phenotype confirmation

Isolates that were tested positive for ESBL by Phoenix were consequently confirmed by Double Disc Synergy Test (DDST) as previously described [17, 18]. Briefly, synergy was determined between a 20/10 μg disc of amoxicillin-clavulanate (BD- Sensi Disc™) and 30-μg disc of ceftazidime and ceftriaxone (BD- Sensi Disc™), placed onto Mueller–Hinton agar (Oxoid Ltd., Basingstoke, Hampshire, England) inoculated with a microbial suspension of 0.5 McFarland turbidity, at a distance of 15 mm apart from the edge of the amoxicillin-clavulanate disc. The cefoxitin (30 μg, BD- Sensi Disc™) disc was placed in any available space remaining on the plate. Extension of the edge of the exhibition zone by > 5 mm towards the disc of amoxicillin-clavulanate disc, together with susceptibility to cefoxitin was interpreted as positive for the ESBL production [18].

### Molecular genotyping of ESBL genes

DNA was extracted from bacterial cultures using QIAamp® UCP pathogen mini Kit (Qiagen, Germany) following manufacturer’s instructions. Extracted DNA was then used to run PCR for seven genes and using previously published primers [19, 20] . The conditions used for ^*bla*^TEM and ^*bla*^SHV reactions were as follows: PCR mixture was made in volume of 20 μl containing 0.5 μM of each primer, 50 ng DNA, 1× master mix (Hot star *Taq* plus master mix (Qiagene, Germany)) and DPEC H2O up to 20 μl. The reaction was amplified in GeneAmp* PCR system 9700 thermocycler under the following conditions: 1. Initial denaturation at 96 °C for 5 min.; 2. 32 cycles consisting of denaturation at 96 °C for 30 s., annealing at 44 °C (^*bla*^ TEM) and at 58 °C (^*bla*^ SHV) for 45 s, and extension for 60 s. at 72 °C; and 3. A final extension cycles at 72 °C for 10 min. Multiplex PCR (MPCR) was performed in a final volume of 30 μl containing 0.23 μM of each primer (^*bla*^ CTX-M-G_(1,2,8,9 &25)_), 50 ng DNA, 1× master mix (Hot star *Taq* plus master mix (Qiagene, Germany) [19]) and DPEC H2O up to 30 μl to screen for ^*bla*^ CTX-M- G (1,2,8,9 & 25) genes. Amplified products were subjected to electrophoresis in 1.2% agarose (Agarose- LE, Ambion®, USA), stained with ethidium bromide (Promega, Madison, USA) and visualized using Bio-Rad gel doc system (Bio - rad, Gel Doc ^tm^ XR System 170–8170, Canada).

### Clustering of ESBL-positive isolates

An agglomerative hierarchical algorithm was used to derive a cluster analysis dendrogram to establish the relationship between individual *E. coli* (*n* = 95) and *Klebsiella pneumoniae* (*n* = 13) isolates based on the presence and absence of 7 genes (^*bla*^TEM, ^*bla*^SHV, ^*bla*^ CTXM -G 1, ^*bla*^ CTXM -G2, ^*bla*^ CTXM -G8, ^*bla*^ CTXM -G9 & ^*bla*^ CTXM –G25), whichare reported in the literature to encode for ESBL. The scores ‘1’ and ‘0’ were given for the presence and absence of bands respectively [21–23]. The data obtained by scoring of the genetic profiles of different ESBL genes were subjected to cluster analysis, and hierarchical cluster dendrogram was created using Past software version 1.91 [24]. A similarity matrix values were used for cluster analysis.

### Data analysis

Data were introduced into Microsoft Excel 2010 (Microsoft Corporation, New York, USA) to generate figures and run initial analysis and further statistical analysis were done using SPSS statistics 24 (Statistical Package for the Social Science; SPSS Inc., Chicago, IL, USA). Relation between gene type and resistance of each antibiotic was cross-tabulated using non-parametric Kappa statistics; on the other hand, relation between gene type, nationality and age grouping was calculated using Pearson chi-square test. Probability value (*P* value) less than 0.05 was considered statistically significant. Past software, version 1.91, was used to construct hierarchical clustering dendrogram and Jacquard’s coefficient was applied to generate the similarity values for generation of the cluster analysis [24].

## Results

### Demography of the study population

The demographic profile of the studied population is summarized in Table 1. Sixteen percent (*n* = 34) of samples were collected from males compared to 83.% (*n* = 167) from females (0–15 years of age), with Male to female ratio of approximately 1:5. ESBL producing Enterobacteriaceae were more prevalent among Qataris 46 (22.8%), Egyptians 37 (18.4%), Indians 27 (13.4%), and to lesser extent in Pakistani 21(10.4%). Most of the ESBL was detected among children between 0 and 5 years of age, *n* = 142 (70.6%).

**Table 1:** Demographic profile of the study population (*n* = 201) with ESBL UTI in the State of Qatar

Gender	Total number/percentage	Nationality	
		Qatari	Non Qatari (*n*^a^=24)
Male	34 (16.9%)	5 (2.5%)	29 (14.42%)
Female	167(83%)	41 (20.4%)	126 (62.7%)
Total no./percentage	201 (100%)	46 (22.9%)	155 (77.11%)
Age group (years)			
< 2	60 (29.9%)	7 (36.8%)	53 (26.4%)
2–5	82 (40.8%)	18 (9%)	64 (31.8%)
6–15	59 (29.4%)	20 (10%)	39 (19.4)

^a^Represent the number of nationalities tested

### Etiology of ESBL-associated UTI infections

Out of 635 Enterobacteriaceae positive urine cultures, 201 (31.7%) were found to be ESBL producing bacteria. *E. coli* species was the most prominent with prevalence rate of 83% (*n* = 166), followed by *Klebsiella pneumoniae* 11% (*n* = 22) and the rest 6% included *Citrobacter koseri*, *Enterobacter cloacae*, *Serratia marcescens*, *Citrobacter amalonaticus*.

### Phenotypic resistance profile of ESBL isolates

**Fig. 1:**
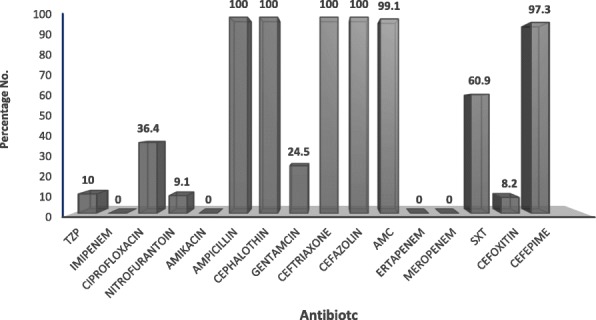
Antimicrobial resistance profile of 201 ESBL producing bacteria isolated from children (age 0 to 15 years) with UTI. Isolates were tested for antibiotics resistance against 16 clinically relevant antibiotics using phoenix NMIC/ID-5 panel (BD Biosciences, Heidelberg, Germany). The figure depicts the percentage of isolates with resistance to each of the antibiotics. TZP: piperacillin/tazobactam; SXT: trimethoprim/sulfamethoxazole; AMC: Amoxicillin/clavulanic acid

Antibiotics resistance profile of ESBL-producing pathogens is depicted in Fig. 1. All ESBL-producing isolates showed 100% resistance to ampicillin, and to all cephalosporins including cephalothin, cefazolin, ceftriaxone and cefepime. Low resistance was recorded to carbapenems. Resistance ranged between 2.5% to meropenem, ertapenem and 10% to imipenem. Among the β-lactam/β-lactamase inhibitor combinations, 9% were resistant to piperacillin/tazobactam, whereas 99% were resistant to amoxicillin/clavulanic acid. Regarding aminoglycosides, all the isolates were susceptible to amikacin, and 24.4% of which were resistant to gentamicin. The resistance prevalence to other classes of antibiotics namely, cefoxitin, nitrofurantoin, trimethoprim/sulfamethoxazole and ciprofloxacin was 19.4, 13, 59.7 and 36%, respectively.

### Molecular genotyping profile of ESBL isolates

**Fig. 2:**
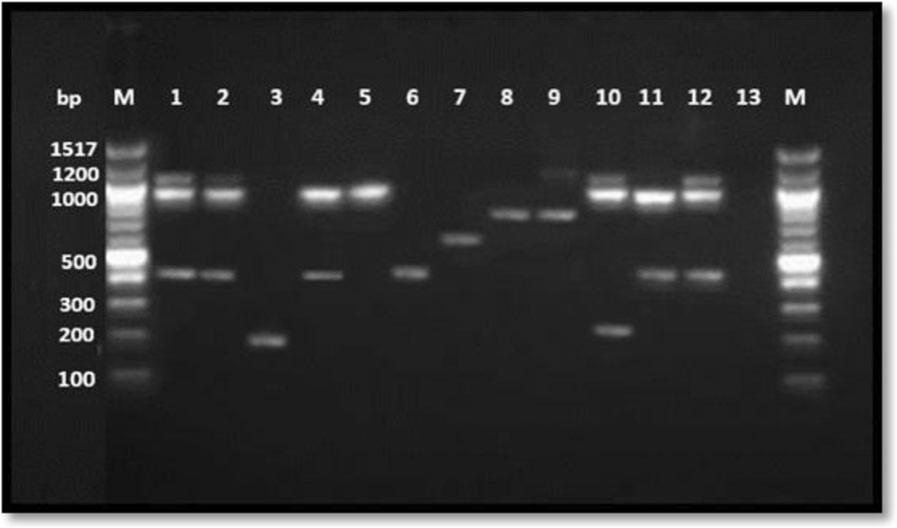
Detection of ^*bla*^SHV, blaTEM and ^*bla*^CTX-M-G (1,2,8,9, &25) antibiotic resistance genes in 110 ESBL Enterobacteriaceae pathogens isolated from children with UTIs. Representative samples are shown. Multiplex PCR was performed for detection of CTX-M groups while monoplex PCR was used for detection of TEM and SHV. The amplification products of each isolate were run on the same lane for detection of bla genes. Lane 1: ^*bla*^TEM, blaSHV& ^*bla*^CTX-MG1; Lane 2: ^*bla*^TEM, ^*bla*^SHV & bla CTX-M-G1: Lane 3: ^*bla*^CTXM-G9; Lane 4: ^*bla*^SHV & ^*bla*^CTXM-G1; Lane 5: ^*bla*^SHV; Lane6: ^*bla*^CTX-MG1; Lane 7: ^*bla*^CTXM-G2; Lane 8; ^*bla*^CTXM-G8, Lane 9: ^*bla*^TEM &^*bla*^CTXM-G8; Lane 10, ^*bla*^ TEM, ^*bla*^SHV &^*bla*^CTXM-G8; Lane 11: ^*bla*^SHV & ^*bla*^CTXM-G1; Lane 12: ^*bla*^NCTC 13,351 *E. coli* Positive control for ^*bla*^TEM, NCTC 13368 *K.* pneumonia positive control for ^*bla*^SHV, NCTC 13461 *E. coli* positive control for ^*bla*^CTX-MG1: Lane 13: ATCC 25922 *E. coli* negative control

**Fig. 3:**
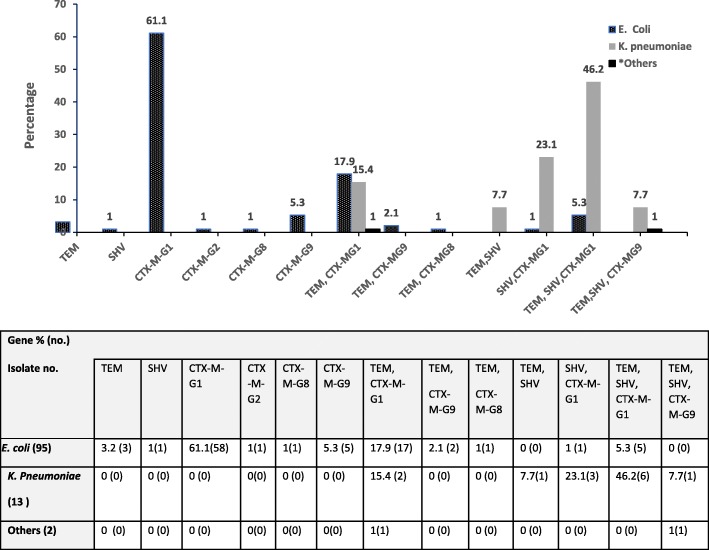
Distribution of bla genes among ESBL Enterobacteriaceae obtained from urine samples of children with lower urinary tract infection

110 ESBL producing bacterial isolates representing 95 *E. coli*, 13 *K. Pneumonia*, one *Citrobacter koseri* and one *Enterobacter cloacae* were randomly selected and characterized with PCR for genes encoding resistance (Fig. 2). Of these, the highest resistance (*n* = 65, 59%) was encoded by ^*bla*^ CTX-M genes: ^*bla*^ CTX-MG1 (89.2%), ^*bla*^ CTX-MG9 (7.7%), and ^*bla*^ CTX-MG2 (0.9%) and ^*bla*^ CTX-MG8 (1.5%). ^*bla*^ TEM and ^*bla*^ SHV genes were detected in 2.7 and 0.9% of the isolates, respectively. 37.3% of bacteria harbored multiple *bla* genes (≥ two genes). Two *bla* genes were detected in 20 (18.2%) *E. coli* and 3 (3.2%) of *K. pneoumoniae* isolates. 77% of *K. pneoumoniae* and 6.3% of *E. coli isolates were harboring* three *bla* genes. While majority of the ESBL *E. coli* resistance was encoded by one gene, ^*bla*^CTX-M-G1 58 (61.1%), resistance in *k. pneoumoniae* isolates was encoded by ^*bla*^SHV, ^*bla*^TEM and ^*bla*^CTX-MG1 genes (46.2%) concurrently (Fig. 3). ^*bla*^CTX-M-G25 was not detected in any isolate.

### Correlation between phenotypic and genotypic profiles

The antibiotic resistance outcomes (resistance or susceptible [i.e., binary]) were cross- tabulated with the six detected ESBL genes using kappa statistics (Table 2). A significant association (*p* < 0.05) were found between the presence of TEM, nitrofurantoin and trimethoprim/sulfamethoxazole; SHV and nitrofurantoin; CTXM-G2 and piperacillin/tazobactam. The presence of individual ESBL genes was not significantly different (*P* > 0.05, Pearson Chi- square test) by nationality or age.

**Table 2:** Measure of agreement between resistance of antibiotics and presence of ESBL genes by cross tabulation Kappa statistics

Antibiotic	Number of resistance / Kappa significant value					
	TEM	SHV	CTXM-G1	CTXM-G2	CTXM-G8	CTXM-G9
Piperacillin/Tazobactam	11/0.186	11/0.253	11/0.5	11/0.003	11/0.634	11/0.328
Ciprofloxacin	40/0.06	40/0.654	40/0.401	40/0.2	40/0.9	40/0.9
Nitrofurantoin	12/0.021	12/0.000	12/0.825	12/0.7	12/0.074	12/0.3
Gentamicin	28/0.2	28/0.843	28/0.08	28/0.4	12/404	12/0.9
Trimethoprim/Sulfamethoxazole	67/0.02	67/0.727	67/0.2	67/0.08	67/0.75	67/0.396
Cefoxitin	11/0.180	11/0.538	11/0.7	11/0.057	11/0.057	11/0.8

### Clustering and similarity of ESBL-positive isolates

**Fig. 4:**
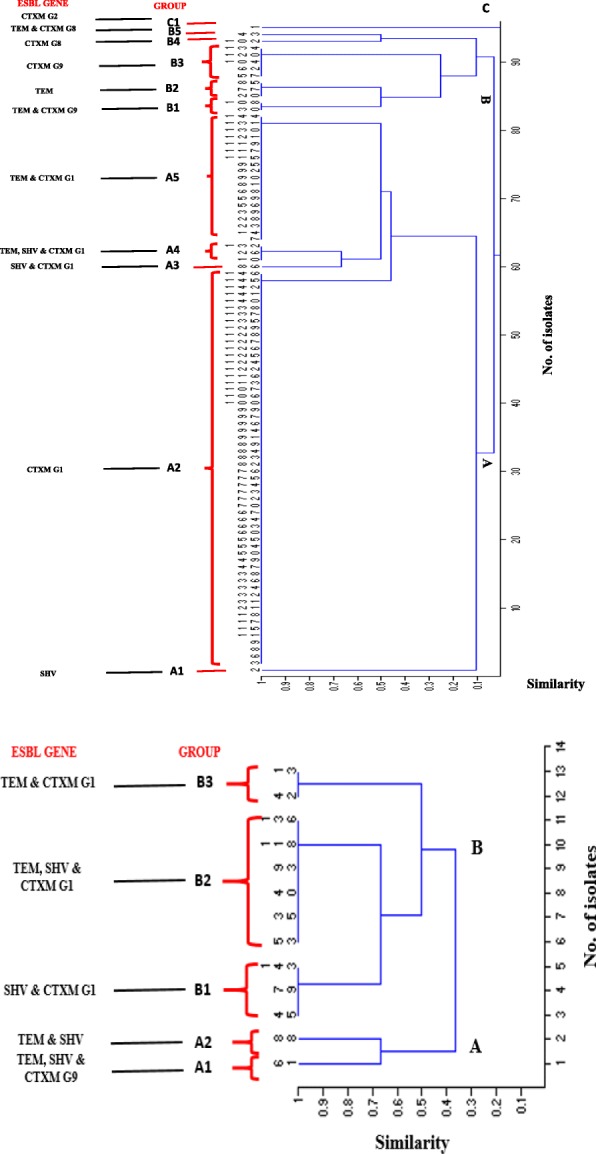
Agglomerative hierarchical algorithm illustrating the similarity of *E. coli* (**a**) and *K. pneumoniae* (**b**) based on the presence and absence of ESBL genes, using past software version, 1.91 and Jaccard’s coefficient. A similarity value = 0 indicates dissimilarity between organisms, while similarity value of 1 means the organisms are identical or very close to each other

Cluster analysis was used to study similarity among individual *E.coli* (*n* = 94) and *K. pneumoniae* (*n* = 13) isolates according to presence and absence of 7 genes (^*bla*^ TEM, ^*bla*^ SHV, ^*bla*^ CTXM -G 1, ^*bla*^ CTXM- G2, ^*bla*^ CTXM G8, ^*bla*^ CTXM G9 & ^bla^ CTXM G25). The *E. coli* positive isolates were distributed into one of the three main branches A, B and C (Fig. 4a), then sub grouped into 11 clusters (A1, A2, A3, A4 A5, B1, B2, B3, B4, B5 and C1). Most of the *E. coli* isolates clustered in A2 (61.7%) which includes only CTXM-G1 enzyme.Around 20.2% clustered in A5 (which includes a combination of TEM and CTXM-G1 enzymes. On the other hand, *K. pneoumoniae* were distributed into one of the two main branches A and B (Fig. 4b), then sub grouped into five clusters (A1, A2, B1, B2 and B3), with the main cluster being B2 (46.2%) which represent combination of TEM, SHV and CTXM-G1 type enzymes.

## Discussion

Out of a 635 Enterobacteriaceae isolated from urine samples obtained from a cohort of children with UTI, 201 (31.7%) were found to be ESBL. *E. coli* species was the most prevalent representing 83% (*n* = 166) of the isolates, followed by *K. pneumoniae* which represented 11% (*n* = 22). This is in agreement with other studies including a recent one from Sri Lanka [25] in which *E. coli* and *Klebsiella* species represented 86.8 and 13.1% of UTI infections among adult patients. Expectedly, ESBL was more predominant among females than males with a ratio of 4.9 to 1. It has been frequently reported thatUTIs occur far more frequently in girls than in boys during the first few months of life, presumably due to the shorter length of the female urethra [26, 27]. The most affected group of our study were the children ranging in age from 0 to 5 years (70.7%), wherecolonization with *E. coli* and enterococci is known to diminish after 5 years of age [26].

Molecular genotyping of ESBL-positive isolates showed that the highest resistance was due to the presence of ^*bla*^ CTX-M genes (59%), particularly ^*bla*^ CTX-MG1 (89.2%). ^*bla*^ TEM and ^bla^ SHV genes contributed to only 2.7 and 0.9% of ESBL resistance, which is consistent with previous reports from the region and around the globe [15, 28–31]. The high occurrence of ^*bla*^ CTX-MG1 gene among pediatric population suggests high dynamic transmission-ability of the plasmid-carried gene, that contributes to the dissemination of CTX-M enzymes to the community and could cause extra horizontal transmission in healthcare facilities [32]. In busy health care facilities, especially in developing countries, the rapid turnover of patients to accommodate multiple patients’ treatment could lead to breaching of infection control measures, such as compliance with hand hygiene, which could lead to this horizontal transmission of resistant bacteria from one patient to another. Unfortunately, we could not compare our findings to others from the region due to the scarcity of published data. However, it has been globally reported that the predominant genotype of ESBL-producing *E. coli* and *K. pneumonia* has changed from TEM and/or SHV to CTX-M-1 since 2006 [33, 34], which seems to be the trend in Qatar. Interestingly, our results indicated that three ^*bla*^ genes (^*bla*^SHV, ^*bla*^TEM and ^*bla*^CTX-MG1/ ^*bla*^CTX-MG9) were concurrently detected in 77% of *K. pneoumoniae isolates*, whereas only 6.3% of the *E. coli* isolates had combinations of the three genes (Fig. 3). On the other hand, two out of seven ^*bla*^ genes were simultaneously detected in 18.2 and 3.2% of *E. coli* and *K. pneumonia,* respectively. These results indicate different genotypic profile of ESBL resistance between *E. coli* and *K. pneumoniae* in urine samples which might indicate a high transfer of genes among *K. pneumoniae* than *E. coli*. Concerning the cephalosporins, all ESBL-producing isolates showed 100% resistance to ampicillin, cephalothin, cefazolin, ceftriaxone and 97.3% resistance to cefepime. This finding coincided with a study carried in intensive care unit (ICU) among adult patients at HMC, Qatar, on respiratory tract and blood samples [35]. No resistance in ESBL-producing isolates were observed against amikacin, ertapenem, meropenem and imipenem. Among the β-lactam/β-lactamase inhibitor combinations, 10% were resistant to piperacillin/tazobactam, whereas 99.1% were resistant to amoxicillin/clavulanic acid. The clinical isolates presented 24.5% resistance to gentamicin compared with 100% susceptibility to amikacin among aminoglycosides. Similarly, these findings concur with the findings from the aforementioned study in ICU patients, [35] with an exception of piperacillin/tazobactam resistance which was reported at 22% in ICU adult patients compared to 10% in pediatrics with UTI. Resistance to piperacillin/tazobactam and ciprofloxacin were significantly lower in our study (10 and 36.4% respectively) compared with another one from Kingdom Saud Arabia (KSA) in a hospital at Dammam-Eastern province, where resistance reached63.2 and 84.6%, respectively [30]. Authors attributed high resistance to these antibiotics due to the misuse of ciprofloxacin and restriction of aminoglycosides among adult in Dammam hospital. Whereas, ciprofloxacin is restricted for use among adult patients at HMC and very rarely used in pediatric population (personal communication, 2017).

The resistance to oral antibiotics namely, nitrofurantoin, trimethoprim/sulfamethoxazole and ciprofloxacin was found to be 9.1, 60.9 and 36.4%, respectively. Obtained results were comparable to those observed in the Middle East region [27], which indicate rapid dissemination of multidrug resistance bacteria in the communities [15, 36]. Worldwide, previous studies have reported higher prevalence of ESBL resistance in Europe compared to United States (US), but lower than South America and Asia [37]. Our results indicate that the patterns of ESBL resistance in Qatar, at least in pediatric population, is more similar to Asia. It is worth noting that 80% of population in Qatar is expatriates; arriving mostly from countries in South East Asia. This could partially explain our findings. Cumulatively, our findings mandates the establishment of the antimicrobial stewardship program and formulation of guidelines for empirical use of prescription of antibiotics for UTI infections among pediatric population.

We found that the majority of ESBL (156, 77.6%) producers were multidrug resistant (resistant to 3 or more different classes of antibiotics), with the most common resistance pattern being to amoxicillin-clavulanate, ampicillin, ciprofloxacin, Sulfamethoxazole/trimethoprim in addition to cephalosporines. Plasmids encoding ESBL resistance often carry multiple genes conferring multiple resistance, thus simultaneous resistance to fluoroquinolones, aminoglycosides, tetracyclines, and trimethoprim sulfamethoxazole [12]. Our study revealed a significant association (*p* < 0.05) between the presence of TEM, nitrofurantoin and trimethoprim/sulfamethoxazole; SHV and nitrofurantoin; CTXM-G2 and piperacillin/tazobactam resistance. Thus, in our study, the use of extended-spectrum cephalosporins, ciprofloxacin, aminoglycosides (gentamicin) and trimethoprim/sulfamethoxazole is compromised, leaving carbapenems as the therapeutic option for severe infections caused by ESBL producers. In agreement with our findings, [38] found that plasmid-mediated quinolone resistance has been associated with ^bla^ CTX-M genes, where genes conferring resistance to aminoglycosides and tetracycline and other ^*bla*^ genes have been found on the same plasmids as the ^bla^ CTXM [39]. Isolates in this study were all from pediatric patients in the community who attended emergency setting This highlights the therapeutic challenges posed by ESBL producers, particularly in the UTI treatment of community-onset [40].

Using seven genes to run agglomerative hierarchical algorithm analysis [21–24] our study revealed eleven clonal clusters among the 94 tested *E. coli* isolates. Clonally related strains of cluster A2 were responsible for the predominant UTI in pediatrics population 67.1% (58/94), which produce only CTXM-G1 type enzyme, followed by A5 (20.2%), which produce a combination of TEM and CTXM-G1type enzymes. Five clonal clusters were detected among *K. pneumoniae* with the main cluster B2 producing concurrently a combination of three enzyme types SHV, TEM and CTXM-G1. Whole plasmid sequencing for representative isolates from each cluster can be very helpful to better understand the relatedness and typing differences between the clusters and elucidating further information on the mechanism of resistance in these isolates. Unfortunately, similar molecular analysis of ESBL bacteria are lacking in the surrounding country. This limit our understanding of the trends and distribution of the strains, noting that more than 80% of Qatari population are expats that arrive from MENA region and South East Asia.

## Conclusions

Although ESBL-producing members of Enterobacteriaceae have been reported in all Arabian Gulf region, very limited data is available about the genetic makeup encoding for such resistance. This is the first study among pediatric population in the state of Qatar that demonstrate the correlation between genetic and phenotypic profile of ESBL producing Enterobacteriaceae. The study highlights an escalated problem of ESBL resistance in Enterobacteriaceae causing UTI in pediatric population in Qatar. Resistance was predominantly mediated by CTX-MG1-type enzymes. The use of cephalosporins, aminoglycosides (gentamicin) and trimethoprim/sulfamethoxazole is compromised in Qatar among pediatric population with UTI, leaving carbapenems and amikacin as the therapeutic option for severe infections caused by ESBL producers. The negative impact of extensive use of carpabenemes could lead to carbapenamase resistant Enterobacteriaceae. This mandates further epidemiological studies and implementation of regulatory programs to reduce spread of ESBL producing Enterobacteriaceae in the community. Further next generation sequencing (NGS) studies are necessary for a more comprehensive analysis of ESBL variants.

## Abbreviations

CP: Cerebral palsy
DDST: Double disc synergy test
ESBL: Extended-spectrum beta-lactamase
GCC: Gulf cooperation council
GNB: Negative bacteria
HMC: Hamad medical corporation
MRC: Medical research centre
NGS: Next generation sequencing
UTI: Urinary tract infection
